# Screening a repurposing library, the Medicines for Malaria Venture Stasis Box, against *Schistosoma mansoni*

**DOI:** 10.1186/s13071-018-2855-z

**Published:** 2018-05-15

**Authors:** Valérian Pasche, Benoît Laleu, Jennifer Keiser

**Affiliations:** 1 Department of Medical Parasitology and Infection Biology, Swiss Tropical and Public Health Institute, P.O. Box, CH-4002, Basel, Switzerland; 2 University of Basel, P.O. Box, CH-4003, Basel, Switzerland; 3 Medicines for Malaria Venture (MMV), PO Box 1826, 20, Route de Pré-Bois, 1215 Geneva 15, Switzerland

**Keywords:** Schistosomiasis, Anthelminthics, *Schistosoma mansoni*, Drug discovery

## Abstract

**Background:**

The development of new treatments against schistosomiasis is imperative but lacks commercial interest. Drug repurposing represents a suitable strategy to identify potential treatments, which have already unblocked several essential steps along the drug development path, hence reducing costs and timelines. Promoting this approach, the Medicines for Malaria Venture (MMV) recently distributed a drug repurposing library of 400 advanced lead candidates (Stasis Box).

**Methods:**

All 400 compounds were initially tested in vitro against the larval stage of *Schistosoma mansoni* at 10 μM. Hits progressed to screening on adult worms and were further characterised for IC_50,_ cytotoxicity and selectivity. Ten lead compounds were tested in mice harbouring a chronic *S. mansoni* infection.

**Results:**

Eleven of the 37 compounds active on the larval stage were also highly active on adult worms in vitro (IC_50_ = 2.0–7.5 μM). IC_50_ values on adult *S. mansoni* decreased substantially in the presence of albumin (7.5–123.5 μM). Toxicity to L6 and MRC cells was moderate. A moderate worm burden reduction of 51.6% was observed for MMV690534, while the other 9 compounds showed low activity. None of the in vivo results were statistically significant (*P* > 0.05).

**Conclusions:**

Phenotypic screening of advanced lead compounds is a simple and resource-low method to identify novel anthelminthics. None of the promising hits of the Stasis Box identified in vitro against *S. mansoni* yielded acceptable worm burden reductions in vivo, which might be due to the high plasma protein binding. Since the in vitro hits interfere with different drug targets, they might provide a starting point for target based screening and structure-activity relationship studies.

## Background

The lack of proper sanitation infrastructure worldwide puts more than 700 million people - mostly children - at risk of acquiring schistosomiasis [1–3]. This tropical parasitosis, transmitted by *Schistosoma* spp. trematodes, causes a wide range of severe chronic morbidities that affect the digestive and the urogenital system [2, 4]. Listed as a neglected tropical disease (NTD), schistosomiasis accounts for more than 2.6 million disability-adjusted life years (DALYs) lost [5]. Schistosomiasis has a considerable socio-economic impact in endemic countries, notably by reducing the attendance at school or work place [6, 7]. Schistosomiasis is almost exclusively controlled by preventive chemotherapy with praziquantel. Safe, affordable and an oral treatment, praziquantel presents many advantages [8]. With the global aim to eliminate the disease as a public health problem in the next decade, formalized by the London Declaration on Neglected Tropical Diseases [9], praziquantel treatment coverage will substantially increase over the next years [3, 10]. A major concern is that, in absence of any alternative on the market or advanced candidates in the drug discovery and development pipeline, such intensified use would result in the emergence of drug resistance [11–13]. Hence, in order to meet the long-term goal of elimination, new drug candidates have to be urgently identified [14, 15].

As the return on investment for a new antischistosomal treatment is expected to be very low (or inexistent), drug repurposing is a cost-effective solution to expand the pool of therapeutic candidates. This approach enables the bypassing of certain steps of the development process, which reduces the cost of research and development (R&D) and shortens the “bench to market” period without compromising safety [15, 16]. In this framework and following the same open-access model as the Malaria Box [17] and the Pathogen Box [18], Medicines for Malaria Venture (MMV) selected and compiled a library of 400 compounds. The so-called Stasis Box includes drugs that were stopped at an advanced stage in their clinical development. The reasons for this termination were different for each drug and ranged from lack of efficacy to bankruptcy of the developing company. The availability, the “druglikeness” and the affordability of these molecules were the main criteria used by MMV to build this library. Screening the Stasis Box already identified hits against *Haemonchus contortus* and *Madurella mycetomatis* [19, 20]. It represents therefore a promising and unique repertoire of advanced drugs to test on other organisms particularly those being responsible for neglected tropical or rare diseases. In this study, the activity of the Stasis Box was screened on *Schistosoma mansoni*. These compounds were first tested in vitro on newly transformed schistosomula (NTS). Hits progressed into testing on adult worms and in vitro toxicity assays. In vivo studies were performed with selected lead molecules.

## Methods

### Media and compounds

The Stasis Box compounds were compiled for MMV by Evotec (Hamburg, Germany) in 96-wells plates as 10 mM solutions and dissolved in 10 μl pure DMSO. The plates were stored at -80 °C until use. Stock solutions (1 mM) in M199 medium were prepared for the in vitro assays. For IC_50_ determination on adult worms, cytotoxicity assays and in vivo studies, MMV690732 and MMV690787 were purchased from Adooq Bioscience (Irvine, USA), MMV690596, MMV690599 and MMV690646 were purchased from Bio-Techne (Minneapolis, USA), MMV690466 and MMV690765 were purchased from SanBio BV/Cayman (Uden, The Netherlands), MMV690684 was purchased from Selleck Chemicals (Houston, USA), MMV690534 and MMV001539 were purchased from Sigma-Aldrich (Buchs, Switzerland). For NTS transformation and maintenance of the parasites, Hank Balanced Salt Solution 1X (HBSS), M199 medium and RPMI 1640 were purchased from Gibco (Waltham MA, USA). Penicillin/Streptomycin 10’000 U/ml and inactivated foetal calf serum (iFCS) were purchased from Bioconcept AG (Allschwil, Switzerland). For cytotoxicity assays, rat skeletal myoblast L6 cells were grown in RPMI supplemented with FCS and L-glutamin (Sigma-Aldrich). Podophyllotoxin (PTT) was purchased from Sigma-Aldrich and stock solutions (5 μg/ml) were prepared in L6 cells medium.

### *Schistosoma mansoni* adult worms and schistosomula

The *S. mansoni* (Liberian strain) life-cycle, is maintained in-house at the Swiss Tropical and Public Health Institute (Swiss TPH), as described before [21]. *Biomphalaria glabrata* were infected with 6 to 8 *S. mansoni* miracidia. They were kept in pond water under natural light, temperature and humidity conditions until the infectious stage, the cercariae, started to shed. The cercariae were mechanically transformed to schistosomula, the NTS, using a procedure adapted from Milligan & Jolly [22]. NTS were incubated (37 °C, 5% CO_2_) until use for 12 to maximum 24 h in M199 medium supplemented with FCS and antibiotics. Adult *S. mansoni* of both sexes were collected by dissecting the intestinal veins of mice euthanized 7 weeks post-infection. All the adult worms recovered were incubated for maximum one week until use in RPMI medium supplemented with FCS and penicillin-streptomycin.

### In vitro assays

For in vitro screening on NTS, the parasite suspension was adjusted to 50 NTS/100 μl in supplemented M199 medium and added to the drug dilutions in 96-wells plates (Eppendorf AG, Hamburg, Germany). NTS were initially exposed to a drug concentration of 10 μM (0.1% DMSO). Each drug was tested in duplicate.

Hit compounds identified on NTS progressed into testing on adult worms. Females, males and pairs (3 to 5 worms per well) were exposed to the drug dilution in supplemented RPMI medium. The assays were performed in duplicate in 24-wells plates (Eppendorf AG, Hamburg, Germany). IC_50_ values were determined for compounds that demonstrated a high activity against NTS and adults (effect ≥ 75% at 10 μM after 72 h). Each assay on NTS was performed in triplicate with serial drug dilutions (1:2, range: 10–0.16 μM) and repeated once. Similarly, the IC_50_ values on adult worms were determined after incubating the worms for 72 h in serial drug dilutions (1:3, range: 33.3–1.23 μM). Each IC_50_ assay was repeated once. These assays were repeated for the lead compounds in presence of albumin (BSA) at the human physiological concentration of 45 g/l (AlbuMAX II, Gibco). For all in vitro assays (NTS and adults), negative controls containing the highest concentration of DMSO were included. Worms incubated with praziquantel served as positive controls. The parasites were evaluated under an inverted optical microscope over 3 days after drug exposure. Their movement and morphology were assessed and scored as described previously [23, 24].

### Cytotoxicity assays

Rat skeletal myoblast L6 cells (2 × 10^4^ cells/ml) were exposed to serial dilutions of the 11 lead compounds (1:3, range: 0.37–90 μM). Podophyllotoxin (PPT, 1:3, range: 2 × 10^-4^ –0.05 μg/ml) was used as a positive control. After a 72-hour incubation, resazurin dye (Alamar Blue) was added to the plates. Fluorescence and the cytotoxic concentration (CC_50_) were measured at 530 nm excitation and 590 nm emission wavelength using a SpectraMax M2 (Molecular Device, Sunnyvale CA, USA; Softmax version 5.4.6). Each drug was tested in duplicate and each assay was repeated once.

### In vivo studies

Three weeks old NMRI female mice were used (Charles Rivers, Germany) for in vivo drug efficacy studies. The animals were kept in groups of ten with constant access to food and water. After one week of habituation, mice were sub-cutaneously injected a suspension containing 100 *S. mansoni* cercariae in phosphate-buffered saline (PBS). The drugs (200 mg/kg single dose) were administered seven weeks post-infection by oral gavage to groups of 4 mice. They were dissolved in tap water with Tween80/ethanol (10%, 70:30). Untreated mice (*n* = 10) served as control and were dissected 7 weeks post-infection. Treated mice (*n* = 40) were euthanized with CO_2_ and dissected between 16 to 18 days post-drug administration. The worms were then collected, sexed and counted.

### Statistics

For in vitro assays, the average viability scores between replicates (or individual adult worms) were normalised to the controls and converted into percentage activity (or effect). Drug IC_50_ values (for NTS or adult worms) were calculated by computing different dose-effect values using CompuSyn2 software (ComboSyn Inc., 2007). The linear correlation coefficient (*r*) reflects the experimental fit. In this study, *r*-values > 0.70 were considered acceptable. The mean IC_50_ were considered for analysis only if the values obtained in each replicate did not differ more than 5.5-fold. The selectivity index (SI) of each drug was calculated by dividing the CC_50_ measured on the L6 cells by the IC_50_ measured on the parasites. For in vivo studies, as previously described [25], the worm burden reduction [%] was determined by comparing the worm burden of treated and untreated mice. For statistical significance a Kruskal-Wallis test was employed (R version 3.2.2).

## Results

### In vitro studies

**Fig. 1:**
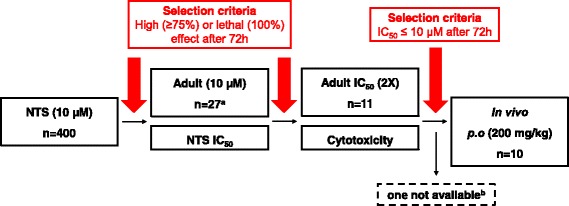
Workflow of the Stasis Box screening on *S. mansoni* worms. ^a^ 26 highly effective hits tested on adults and MMV690466 that showed high activity at 48 h. ^b^ MMV001539 was not available in quantity required for in vivo studies

**Fig. 2:**
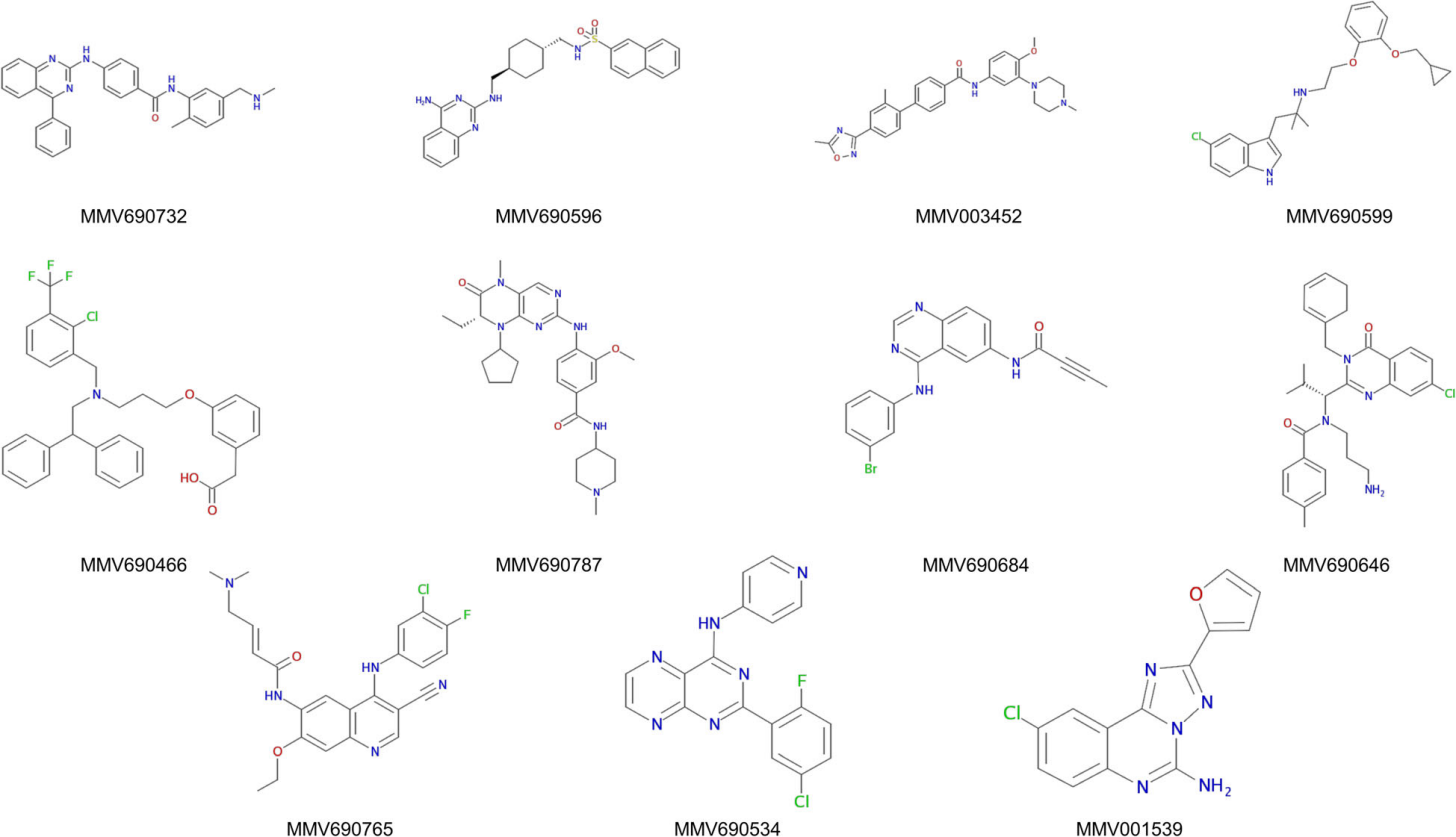
Chemical structures of the Stasis Box lead molecules. Source: ChEMBL

Following incubation for 72 hours, 37 of the 400 drug-like compounds screened were active on NTS at 10 μM (effect ≥ 50%), including 16 that were lethal (effect = 100%) and 10 that were highly active (effect ≥ 75%). Seven of these were fast-acting as they demonstrated a high activity on NTS already after 24 hours (Table 1). The compounds with a moderate effect (< 75%) after 72 h were not investigated further, while the 26 most active ones and MMV690466 - that showed a high effect at the 48 hour time point - proceeded to adult testing (Fig. 1). Sixteen compounds showed only a moderate (effect < 75%) activity against adult worms. On the other hand, 7 compounds displayed a high activity against adult worms and 4 were even lethal (Table 1). Of these, 5 compounds were already highly active against the adult worms after one day incubation period at 10 μM. These 11 compounds were selected as in vitro leads. Compound characteristics and structures are presented in Table 2 and Fig. 2. IC_50_ values calculated 72 h post-incubation ranged from 2 to 7.5 μM on adult worms and 0.5 to 7.2 μM on NTS. In the presence of albumin, a strong decrease in activity of the lead compounds on adult worms was observed (Table 3). The highest activities were observed for MMV003452 and MMV690684 revealing IC_50_ values of 7.5 and 7.7 μM, respectively, denoting a 2-fold increase compared to the values observed without albumin supplementation. The mean IC_50_ values of MMV690596, MMV690599 and MMV690787 were between 10 and 16 μM in the presence of albumin (indicating a 5.4-, 6.1- and 4.3-fold increase, respectively compared to the IC_50_ values without albumin). The remaining 6 compounds showed no activity in the presence of albumin (Table 3).

**Table 1:** Number of hits identified after the initial screen of the Stasis Box at 10 μM

	Evaluation time point		
	24 h	48 h	72 h
NTS hits (effect ≥ 50%)	11	23	37
NTS (effect > 75%)	4	9	10
NTS (lethal)	3	9	16
Adult hits (effect ≥ 50%)	11	9	16
Adult (effect > 75%)	5	4	7
Adult (lethal)	0	2	4

**Table 2:** Stasis Box lead molecules

MMV ID	CHEMBL ID	Name(s)	Indication	Mechanism of action	Target
MMV690732	CHEMBL3545403	XL-139; BMS-833923	Cancer	Smoothened homolog antagonist	Smoothened homolog
MMV690596	CHEMBL1836102	CGP-71683	Obesity, eating disorder	Neuropeptide receptor antagonist	na
MMV003452	CHEMBL15928	GR-127935	Depression	5-HT 1B/1D receptor antagonist	5-HT 1B and 5-HT 1D receptors
MMV690599	CHEMBL88272	RS-17053	Prostate hyperplasia	Alpha 1 adrenoreceptor antagonist	na
MMV690466	CHEMBL59030	GW-3965	Inflammation, melanoma	Agonist of LXR receptor	na
MMV690787	CHEMBL513909	BI-2536	Cancer	Serine/threonine-protein kinase PLK1 inhibitor	Serine/threonine-protein kinase PLK1
MMV690684	CHEMBL91867	CL-387785	Cancer	Epidermal growth factor receptor (EGFR) receptor antagonist + Tyrosine Kinase (TK) inhibitor	na
MMV690646	CHEMBL2111096	CK0238273; SB-715992-S; Ispinesib	Cancer	Kinesin inhibitor	KIF11
MMV690765	CHEMBL607707	EKB-569; Pelitinib	Cancer	EGFR erbB1 inhibitor	EGFR erbB1
MMV690534	CHEMBL238125	SD-208	Cancer, chronic pulmonary obstruction	TGFb TK inhibitor + receptor antagonist	na
MMV001539	CHEMBL16687	CGS-15943	Ischemia, stroke	Adenosine A2 receptor antagonist	na

*Abbreviation*: *na* not available

**Table 3:** IC_50_ values on NTS and adult *S. mansoni*, toxicity on L6 and MRC5-cells and selectivity of the 11 Stasis Box lead molecules. Cytotoxic concentration (CC_50_) and selectivity index (SI) are compared to the mean IC_50_ values measured on NTS and adult *S. mansoni* in standard and albumin-enriched medium (45 g/l) over 72 h with 11 Stasis Box lead compounds

Compound MMV ID	NTS IC_50_ (μM)^a^			Adult IC_50_ (μM)^a^			Adult IC_50_ (μM) with albumin^a^			L6 cells		MRC5 cells^d^	
	24 h	48 h	72 h	24 h	48 h	72 h	24 h	48 h	72 h	CC_50_ (μM)	SI	CC_50_ (μM)	SI
MMV690732	29.5	6.3	3.5	4.9	2.9	2.5	62.8	181.0	54.1	2.3	0.9	1.9	0.7
MMV690596	1.1	1.3	0.6	1.0	2.1	2.0	27.5	14.4	11.0	3.5	1.7	1.9	0.9
MMV003452	4.4	2.2	1.2	1.7	3.0	2.7	22.6	12.0	7.5	3.7	1.4	1.9	0.7
MMV690599	10.5	3.5	1.5	2.5	3.3	2.6	23.6	19.1	15.9	3.4	1.3	2	0.8
MMV690466	14.6	3.8	2.1	2.9	2.3	2.0	18.1^b^	89.7	47.6	1.6	0.8	8.4	4.3
MMV690787	38.4^b^	8.1	6.3	7.9	7.2	3.1	39.5	14.4	13.3	na	na	0.1	0
MMV690684	4.0^c^	3.1^c^	0.7	4.9	4.7	3.7	76.8	14.4^b^	7.7	7.0	1.9	7	1.9
MMV690646	3.0^c^	1.1	0.9	3.7	2.2	2.2	32.0	121.8	33.6	na	na	0.2	0.1
MMV690765	914.4^b^	> 1^c^	0.5^b^	4.2	3.4	2.9	651.7	225.9^b^	123.5^b^	11.8	4.1	0.7	0.3
MMV690534	11.8	14.3^c^	7.2^c^	17.4	19.3	7.5	na	na	na	74.5	15.0	12.4	2.5
MMV001539	12.7	11.0^c^	1.7	28.2	12.3	7.0	nd	nd	nd	2.9	0.4	16	2.1

*Abbreviations*: *na* not applicable. *nd* not done

^a^*r*-values ranged between 0.7 and 1.0

^b^These values are based on one replicate only because of an *r*-value < 0.70 was obtained for the second replicate

^c^The IC_50_ values obtained in each of the two replicate differed more than 5.5-fold

^d^The CC_50_ values on MRC5 cells were provided by MMV

The lead molecules were rather cytotoxic, except for MMV690534 that showed, on both mammalian cell lines acceptable parasite-selectivity (SI > 1). When measured on L6 rat myoblast cells MMV690596, MMV003452, MMV690599 and MMV690684 were slightly above the selectivity cut-off while MMV690732 and MMV690466 were slightly below. Although MMV690466 and MMV001539 were not selective towards the worms when measured with L6 cells, the SI was > 1 when measured on MRC5 cells (Table 3).

### In vivo activity

All the compounds tested (*n* = 10) failed to significantly reduce the worm burden in vivo. Eight compounds had no effect on the worm burden in infected mice (worm burden reduction < 36%). Although MMV690534 showed a worm burden reduction slightly above 50%, it was not statistically significant (*P* > 0.05). The mice treated with MMV690646 (Ispinesib) died prematurely and therefore, were not included in the analysis (Table 4).

**Table 4:** In vivo efficacy of the lead molecules from the Stasis Box. Effect on worm burden of a single 200 mg/kg oral dose of nine lead molecules identified after screening the Stasis Box in vitro administered to mice harbouring a 49-day-old adult *S. mansoni* infection

Compound	Mice tested^n^	Mean worm burden ± SD	WBR (%)
MMV690732	4^1^	47.5 ± 24.3	0
MMV690599	3^1^	44.7 ± 29.6	3.2
MMV690787	4^1^	48.3 ± 21.6	0
MMV003452	4^1^	40.5 ± 17.2	12.2
MMV690596	4^1^	52.0 ± 22.7	0
MMV690684	4^1^	41.5 ± 16.5	10.0
MMV690765	3^1^	84.0 ± 56.4	0
MMV690466	4^2^	10.3 ± 6.9	35.9
MMV690534	4^2^	7.8 ± 7.5	51.6
Control^1^	8	46.1 ± 21.9	
Control^2^	2	16.0 ± 19.8	

^n^ Indicates the mice control batch. One mouse died prematurely because of toxic effects of MMV690646 (Ispinesib) and therefore data is not shown

## Discussion

The Stasis Box includes late leads that were abandoned, mainly due to a lack of efficacy against their primary target disease. This library represents therefore a unique repertoire of molecules to test on different organism including *S. mansoni* as the compounds already underwent advanced clinical test phases, which should guarantee satisfactory safety and pharmacological properties.

After screening these drugs in vitro on both larval and adult stages of the parasite, 11 lead molecules revealed strong antischistosomal activity. With the exception of MMV690534 (SD-208) and MMV001539 (CGS-15943), the IC_50_ of the compounds measured on adult worms were already below 10 μM after 24 h, hence the compounds were fast acting (Table 3). Speed of action is an important parameter for defining antischistosomal activity as worms will be exposed only very shortly to high mesenteric vein concentrations of the unmetabolised drug [26]. Speed of action was also already taken into account for drug selection and progression in our previous screenings of an FDA library of approved drugs [25] and of a set of oncology drugs [27]. Although the IC_50_ values measured on NTS after 24 h were very different, they all ranged under the 10 μM cut-off 72 h post-incubation.

The high in vitro activity changed in presence of albumin at the human plasma concentration of 45 g/l. The efficacy of each molecule dropped considerably, notably for MMV690732 (XL-139), MMV690466 (GW-3965) and MMV690646 (Ispinesib) that showed high IC_50_ values ranging from 30 to 55 μM 72 h post-drug exposure in the presence of this protein (Table 3). This finding suggests a strong drug-binding effect of albumin. This finding is consistent with the incapacity of all the lead molecules tested in vivo to significantly reduce the worm burden in infected mice. Hence, the lack of efficacy in vivo might have been caused by a strong drug-binding effect of the host plasma proteins reducing therefore the amount of free drug available to kill the parasite. However, other factors that affect pharmacokinetic processes such as drug metabolism might obviously also play a role.

In order to avoid losing advanced drug leads as potential novel antischistosomal drugs, the hit to lead selection criteria in the present work was less strict than in our previous screenings [25, 27]. For this reason and because the Stasis Box drugs were assumed to have an acceptable safety and pharmacokinetic profile (at least for the relevant therapeutic indication), all compounds moved into in vivo testing despite a higher IC_50_ in the presence of albumin. However, plasma protein binding should be considered among other factors (as clearance, safety, exposure) in the screening cascades, as suggested by Gelmedin et al. [28].

Oncology is a privileged source of drugs to repurpose against schistosomiasis and other NTDs, particularly because of their potency to interfere with conserved signalling pathways that are also involved either in the metabolism or the reproduction of the parasite [11, 15, 29]. For instance, the antischistosomal activity of different protein kinase inhibitors (PTK) has been described previously [28, 30–33]. Also many of the hits identified in our study were developed to target intracellular neoplastic pathways (Table 2). However, one of the disadvantages working with anticancer drugs is obviously toxicity. Although the Stasis Box compounds were selected for their “druglikeness”, the majority of the lead molecules identified were moderately toxic as demonstrated by our cytotoxicity tests, the data provided by MMV, and the death of the mice treated with MMV690646 (Ispinesib). Nonetheless, the identified oncology late leads could serve as starting point for future studies. For example, structure-activity relationship (SAR) studies on these pharmacophores should be conducted to identify less toxic hits.

Our study identified different lead candidates in vitro, which likely act on different targets in *S. mansoni*. For example, we confirmed the in vitro activity of the Polo-like kinase (Plk) competitive inhibitor BI-2536 (MMV690787) on *S. mansoni* [28, 32, 34] targeting SmPlk1 and hence resulting in reproductive impairment of both sexes. MMV690534 (SD-208) might have impaired the reproductive function of the parasite by interfering with the TGF-beta mediated intracellular signalling pathway notably involved in egg production [28, 35, 36]. Aside from reproduction, other functions in *S. mansoni* may have been negatively impacted. For example, epidermal growth factor receptors (EGFR) might have been targeted by antagonists such as MMV690765 (Pelitinib), while MMV001539 (CGS-15943) might have interacted with purinergic receptors. These few examples illustrate the variety of potential targets in *S. mansoni*, notably for kinase inhibitors. In this study, in vitro tests were assessing parasite phenotype (e.g. motility, shape, or colour) after drug exposure. No parameters related to the reproductive function (e.g. egg count) were recorded, which is a limitation of our study given the importance of protein kinases in reproduction.

Considering the good activity in vitro of some of these compounds, it would be worth testing analogues and initiate SAR studies. As the information openly available on these compounds was scarce, manufacturers should be encouraged to provide more information in the public domains. Functional studies are also essential to better characterize potential drug targets and design more effective and selective drugs.

## Conclusions

Despite the fact that no drugs with clear in vivo antischistosomal activity emerged from the Stasis Box screening on *S. mansoni*, different molecules were identified with promising in vitro activity. Together with a better understanding of their potential drug targets, SAR studies could be conducted, particularly taking into account protein binding and in vivo pharmacokinetics. Our findings confirmed that open access libraries such as the Stasis Box are powerful and yet essential tools for drug discovery on NTDs.
